# Recycling of dielectric electroactive materials enabled through thermoplastic PDMS†

**DOI:** 10.1039/d2ra00421f

**Published:** 2022-03-16

**Authors:** Seonghyeon Jeong, Anne Ladegaard Skov, Anders Egede Daugaard

**Affiliations:** Danish Polymer Centre, Department of Chemical and Biochemical Engineering Building 227, Technical University of Denmark 2800 Kgs. Lyngby Denmark adt@kt.dtu.dk; Sino-Danish Center for Education and Research, University of Chinese Academy of Sciences Beijing China

## Abstract

In the green transition, actuators and generators play an essential role in the development of sustainable solutions across a broad range of applications. In this context, dielectric transducers are advocated as one of the most promising solutions in terms of effectiveness, lifetime and running costs. However, they are classically produced as sandwich structures, whereby a cross-linked dielectric material is placed between two compliant electrodes. From a materials consumption viewpoint, this is problematic, since it will inherently result in a loss of material during production as well as inhibit the recycling of expended systems when their life comes to an end. Herein, we present a cleaning method employing surfactants and sonication to remove electrodes from the surface of the dielectric material. By applying a thermoplastic silicone elastomer as the dielectric material, it is possible to reprocess the material by hot-pressing, and to prepare new actuators after the rinsing process. This effectively shows that recycling production scrap, for example, is possible. By comparing the cleaned material with a directly recycled material, it is clear that cleaning removes a critical amount of metals from the material and enables recycling for at least five cycles. Comparatively, a directly recycled material is prone to a high leakage current and premature electronic breakdown after only two cycles. This simple cleaning process, in combination with use of a thermoplastic dielectric material, enables less waste from production as well as the possibility of reclaiming and recycling materials in general.

## Abstract 2

A new recycling method for silver-coated DEAs produced from thermoplastic elastomers. Recycled DEAs retain their dielectric and mechanical properties in five recycling loops in contrast to direct recycling that only permitted a single recycling loop.

## Graphical abstract

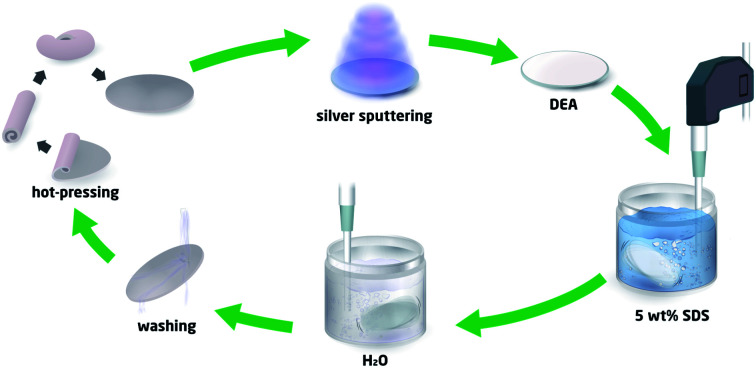

## Introduction

Dielectric transducers (DETs) hold great potential for several applications within the green transition, through both enabling lower power consumption devices such as pumps and soft robotics in actuators (DEAs) and their application in energy-harvesting devices and applications such as generators.^1–4^ However, the implementation of DETs in commodity products raises questions in relation to their sustainability and environmental impacts. DETs are typically prepared as sandwich structures, where an elastomer is cross-linked between two compliant electrodes. Since the central dielectric materials of most DETs are based on covalently cross-linked elastomers, their recycling requires the breaking of covalent bonds to make them reprocessable,^5^ and thus any failure in manufacture currently results in creating waste that cannot be recycled. Existing recycling solutions for plastics, such as in-line shredding and mechanical recycling, efficiently reduce waste from production as well as allow for multiple-use cycles, ultimately reducing the environmental impact of manufacturing. These processes, however, are limited to thermoplastic commodity plastics, whereby acceptable recycled polymer properties can be obtained after recovery, shredding and reprocessing.^6^ Additionally, the recycling process usually builds up contaminants in the polymers, such as traces of heavy metals or impurities that very often cause a negative impact on potential applications^7^ or, in the context of electrical systems, can potentially weaken the electrical properties of the recycled polymers.^8–10^ We recently introduced a physically cross-linked PDMS elastomer that can be processed as a thermoplastic and could potentially enable these more conventional recycling pathways.^11^ However, DEAs are generally very sensitive to imperfections of any kind, since these can promote both mechanical and electrical failure of devices,^12^ which is primarily governed by localised defects within the DEAs,^13–15^ namely electrical treeing, resulting in insulation failure and electrical breakdown.^16,17^ To avoid premature failure, and to increase device reliability, the elastomeric layer must be free from contaminants caused by the recycling process, along with air bubbles, dust particles and moisture. Due to the harsh processes involved in breaking covalent bonds in classical elastomers, thermoplastics would be much more attractive candidates for recyclable DEAs. This could be achieved by establishing, if possible, a commercially feasible reclamation approach that ensures the limited buildup of contaminants during repeated processing.^18^

Herein, we investigate a potential recycling process for DEAs in a realistic configuration, *i.e.* a thermoplastic PDMS elastomer sandwiched between two silver electrodes. Previously, conductive metals such as silver have been demonstrated as compliant electrodes for DEAs, either due to corrugated surfaces or to nanostructuring of the metals.^19–21^ Yet, sputtered silver electrodes sandwiching an insulating thermoplastic elastomer present a large challenge for recycling, since silver impurities in the elastomer present a major challenge in reestablishing the dielectric properties of the pristine elastomer. Small amounts of silver impurities are expected to result in premature breakdown and electrical instability, and so tolerance to such impurities is therefore of utmost interest. In this work, we present a new recycling method for thermoplastic elastomers that is applicable for DEAs. The properly recycled thermoplastic elastomer exhibited almost identical mechanical properties to the pristine one, as evaluated from linear viscoelastic analysis. In addition, all potential changes in dielectric properties are suppressed at low electrical field and no difference can be found. Thus, potentially the presented recycling method may be applied as a general means to recycle the DEAs, including the ones with carbon-based electrodes.

## Experimental

### Materials and methods

Aminopropyl-terminated PDMS (DMS-A31) was purchased from Gelest. Hexamethylene diisocyanate (HMDI, 99%), methanol (99%), 2-amino-4-hydroxy-6-methylpyrimidine (methyl isocytosine, 98%), and silica (SIS6962.0) were purchased from Sigma-Aldrich. Tetrahydrofuran (THF, not stabilised, 99%) was purchased from VWR chemicals. All chemicals were used as received.

### General procedure

The synthesised materials were characterised by a diamond crystal attenuated total reflection (ATR)-equipped Fourier transform infrared (FT-IR) spectrometer (iS50 FTIR spectrometer, NICOLET, US) with a resolution of 4 cm^−1^ and 32 scans per measurement (400–4000 cm^−1^). Nuclear magnetic resonance (NMR) spectra were acquired on an NMR spectrometer (300 MHz, Bruker Avance, UK) in chloroform-d_1_ (CDCl_3_) at room temperature. The molecular weights of the elastomer were estimated by gel permeation chromatography (GPC) on a GPC Solvent/Sample Module (Viscotek GPCmax VE 2001, Malvern, UK), equipped with an evaporative light-scattering detector (PL-ELS 2100, Polymer Laboratories, UK), and two PLgel mixed D columns in series (Polymer Laboratories, UK). THF was used as a mobile phase (flow rate: 1 mL min^−1^) with the addition of 1 wt% of acetic acid. The sample was prepared at a concentration of 1 mg mL^−1^, and the injection volume was 100 μL. Molecular weights were calculated based on narrowly dispersed polydimethylsiloxane (PDMS) standards (PSS, Mainz, Germany).

### Preparation of dielectric elastomer actuator (DEA)

The investigated DEA consisted of TP-PDMS and thin silver electrodes. The following procedures describe the synthesis of the TP-PDMS, and the preparation of the DEA with the TP-PDMS.

#### Synthesis of isocyanate-functionalised ureidopyrimidone (NCO-UPy)

Methyl isocytosine (0.5 g, 3.9 mmol) was loaded into a 25 mL round-bottom flask. Then, HMDI (5 mL, 30 mmol) was added into the reaction flask, and the mixture was stirred for a minute. A reflux condenser was connected to the reaction flask, which was then placed on a hot plate (100 °C). The reaction mixture was stirred for 18 hours. Then the reaction mixture was slowly cooled down to room temperature. The reflux condenser was removed from the reaction flask, and pentane (3 mL) was added to the reaction flask while stirring the reaction mixture. The product was claimed *via* the gravitational filtration of the reaction mixture, and the product was washed with excess pentane. The final product was a white crystal, which was dried at room temperature for 3 days, followed by drying at 50 °C *in vacuo* for 2 days (99% yield). IR spectra (cm^−1^) 3210–3027 (*ν*(N–H)), 3027 (*ν*(C–H) sp^2^ C–H), 2931 (*ν*(C–H) sp^3^ C–H), 2276 (*ν*(N=C=O)), 1698 ((*ν*(C=O) in urea) and 1663 ((*ν*(C=O) in urea). ^1^H NMR spectra (300 MHz, CDCl_3_, *δ*) 13.04 (s, 1H, NH in UPy), 11.79 (s, 1H, NH in UPy), 10.11 (t, 1H, NH in UPy), 5.75 (s, 1H, NH in aromatic ring in UPy), 3.22 (m, 4H, N–CH_2_), 2.16 (s, 3H, Ar-CH_3_), 1.57 (m, 4H, C–CH_2_) and 1.35 (m, 4H, C–CH_2_).

#### Synthesis of TP-PDMS

Aminopropyl-terminated PDMS (DMS-A31, 10.02 g, 0.48 mmol) was dissolved in THF (25 mL) in a 250 mL round-bottom flask. HMDI (69 μL, 0.43 mmol) solution in THF (15 mL) was prepared in a separate vial. Then, HMDI solution was added dropwise into the reaction flask with a syringe over 2 hours while mixing the reaction mixture. The reaction mixture was then stirred for 24 hours at room temperature, followed by the addition of hexamethylene diamine (34.9 mg, 0.3 mmol) to ensure the consumption of all isocyanate functional groups. The reaction mixture was stirred further for another 2 hours, following which it was dried in a rotary evaporator at room temperature and then re-dissolved in THF (10 mL). The solution was precipitated in cold methanol (−32 °C, 100 mL). Then, the precipitate was claimed and dried by a rotary evaporator for 2 hours at 35 °C. A transparent viscous liquid was obtained (yield: 96%). ^1^H NMR spectra (300 MHz, CDCl_3_, *δ*) 3.2 (m, 2H, CH_2_), 2.7 (t, 2H, CH_2_), 1.6–1.4 (m, 2H, CH_2_), 0.5 (m, 2H, CH_2_) and <0.5 (m, 6H, Si-(CH_3_)_2_). The number averaged molecular weight of chain-extended PDMS was calculated by comparing the ^1^H NMR integrals at 3.2 ppm and below 0.5 ppm (110 000 g mol^−1^).

The above product was dissolved in THF (100 mL), and NCO-UPy suspension (6 equiv. mole) in THF (10 mL) was decanted while mixing the solution. The reaction mixture was stirred for 24 hours. Excess NCO-UPy was removed three times by gravitational filtration, resulting in a clear solution as the filtrate. The solution was then dried by a rotary evaporator for 2 hours at room temperature, and it was dissolved in THF (20 mL) again. The precipitation was carried out in cold methanol (−32 °C, 200 mL), where the gel-like precipitate was observed. The methanol was decanted from the flask and the precipitate was carefully washed with cold methanol several times. The above precipitation procedures were repeated three times. The final product was dried by a rotary evaporator at 35 °C for 2 hours, followed by drying at 60 °C for 3 days *in vacuo*. A transparent soft elastomer was obtained (yield: 77%). IR spectra (cm^−1^) 3400–3300 ((*ν*(N–H), hydrogen-bonded in UPy), 3027 (*ν*(C–H) sp^2^ C–H), 2960–2932 (*ν*(C–H) sp^3^ C–H), 2853 (*ν*(C–H) sp^3^ CH2), 1720 (*ν*(C=O) in UPy), 1699 (*ν*(C=O) in urea), 1661 (*ν*(C=O) in urea), 1587, 1527, 1412 (*δ*_s_(C–H) sp^3^ C–H), 1257 (*δ*_s_(C–H) sp^3^ C–H), 1008 (*ν*_as_(Si–O–Si)), 864 (*δ*_as_(C–H) in Si(CH_3_)_2_), 782 (*ν*_as_(Si–C) in Si(CH_3_)_2_) and 700 (*ν*_s_(Si–C) in Si(CH_3_)_2_). ^1^H NMR spectra (300 MHz, CDCl_3_, *δ*) 13.2 (s, 1H, NH in UPy), 11.9 (s, 1H, NH in UPy), 10.1 (s, 1H, NH in UPy), 5.8 (s, 1H, NH in aromatic ring in UPy), 3.3–3.1 (m, 4H, N–CH_2_), 2.2 (s, 3H, Ar-CH_3_) and 1.6–1.3 (m, 12H, CH_2_ in alkyl chains). Molecular weights were obtained from GPC (*M*_n_: 106 000 g mol^−1^, *M*_w_: 175 000 g mol^−1^ (*Đ*: 1.6).

The final product was dissolved in THF (20 mL) in a 50 mL speed mixer cup. Then, 14 wt% silica was added, followed by speed mixing at 3000 rpm for 10 minutes. The TP-PDMS solution was dried at room temperature for 3 days, followed by drying at 60 °C *in vacuo* for 3 days. Then, TP-PDMS was hot-pressed (140 °C, 40 bar) within a 1 mm-thick mould for 2 minutes.

### Preparation of the (TP-PDMS)-based DEA

The TP-PDMS was punched out into a disk shape (diameter: 25 mm). The precise thickness of the TP-PDMS disk was measured 10 times by a coating thickness meter (PFN-52D, MEGA-CHECK Pocket, Germany), and the average thickness was reported. The specific amount of silver (0.024 wt%) was sputter-coated on the top area of the disk, using a rotary pumped coater (Q150R Plus, Quorum, UK) connected to a rotary vacuum pump (Duo 6, Pfeiffer, Germany). The amount of silver was controlled to obtain a thickness of the silver layer of around 10 nm using a PTFE-functionalised PET film as a masking film on the top area of the TP-PDMS disk.

Accumulation of the silver electrode (P1_1 and P1_2) The DEA was prepared from the pristine TP-PDMS disk mentioned from the previous chapter. Then, the DEA was heated for a minute in an oven at 140 °C, followed by randomly distributing the silver within the elastomer *via* a two-roll mill (MW-06, Dijatec, Netherlands). The procedure was repeated until the overall colour of the recycled TP-PDMS was homogeneous, typically 10 times. The recycled TP-PDMS was hot-pressed within a 1 mm-thick disk-shaped mould. The sample was denoted as P1_1. When the above procedure was repeated twice, the recycled elastomer was denoted as P1_2 and so forth.

Preparation of recycled elastomer (P2_1, P2_3 and P2_5) Sodium dodecyl sulphate (SDS, 500 mg) was dissolved in deionised water (10 mL) in a vial, in order to prepare a 5 wt% SDS solution. The DEA was added to the SDS solution, which was then ultra-sonicated by an ultrasonic processor (200W, 24 kHz, UP200S, Hielscher Ultrasonics GmbH, Germany) for an hour. The DEA was transferred into another vial with deionised water, and it was ultra-sonicated for 10 minutes. Afterwards, the DEA was taken out from the vial and washed with running deionised water. The reclaimed elastomer from the DEA was dried at 60 °C *in vacuo* for a day. After the above cleaning procedures, the reclaimed TP-PDMS was folded multiple times, and it was hot-pressed in a 1 mm-thick disk-shaped mould at 140 °C, 20 bar for 2 minutes.

The precise thickness of the hot-pressed film was measured by the previously mentioned method. Then, the silver (0.024 wt%) for the PDMS TPE was sputter-coated again. To evaluate recyclability, the above-mentioned recycling procedures were repeated up to five times on the same TP-PDMS and denoted as P2_1, P2_3 and P2_5 for 1, 3 and 5 times, respectively, for the recycled TP-PDMS.

### Evaluation of recyclability

The impact of recycling the DEA on characteristic elastomer properties was evaluated by the following methods:

#### Analysis of colour change

The 1 mm-thick pristine and recycled TP-PDMS films were punched out into small disks (diameter: 3 mm) and placed in an acrylic 96-well plate (CORNING, US). To monitor the optical properties of the recycled TP-PDMS, ultraviolet-visible (UV-Vis) spectra were acquired *via* a microplate reader (POLARstar OMEGA, BMG Labtech, Germany), where the absorbance of the elastomers was monitored from 220 nm to 1000 nm.

#### Analysis of rheological properties

The 1 mm-thick pristine and recycled TP-PDMS films were punched out into disks (diameter: 20 mm). The samples were then inserted in the rheometer (Discovery HR-1, TA instrument, US), equipped with two parallel plate fixtures (diameter: 20 mm). The gap between the two parallel plates was controlled after loading the sample to provide perfect contact between the plates and the samples, resulting an axial force of 2–3 N. Viscoelastic properties of the samples were measured with the oscillatory frequency sweep method (10^2^–10^−2^ Hz, 3% strain) within the linear viscoelastic region.

#### Analysis of dielectric characteristics

The pristine and recycled TP-PDMS films were hot-pressed (140 °C, 10 bar) for 2 minutes. The thickness of the samples was measured 10 times and averaged as mentioned previously, and silver was sputter-coated on both sides of the films at a diameter of 20 mm (thickness: 20 nm). Two gold electrodes (diameter: 20 mm) were placed onto the samples' electrodes. Then, they were placed on the sample cell of a Novocontrol Alpha-A high-performance frequency analyser (Concept 40 Top Class system), to analyse the dielectric relaxation of the samples in the range 10^6^–10^−1^ Hz in an electrical field measuring ∼1 V. The measurements were carried out at room temperature.

#### Analysis of leakage currents

The pristine and recycled TP-PDMS films were hot-pressed under the same condition as above. The leakage current of the samples was measured with a high-voltage leakage current test system (PK-SPIV17, PolyK Technologies, US), connected to a high-voltage power supply (PS 350, Stanford Research System, US), and an electrometer (6517A, Keithley, US) to measure the leakage current. Voltage was fixed at 10 V μm^−1^, and the measurements were carried out for 2 minutes unless the sample underwent electrical breakdown, after which the experiment was stopped.

#### Actuation tests

The pristine and five-times recycled TP-PDMS (P0 and P2_5) were hot-pressed (140 °C, 40 bar) inside a 1 mm-thick mould for 2 minutes. Subsequently, the films were hot-pressed (120 °C, 20 bar), without the use of a mould, for 1 minute. The thickness of the films was measured 10 times by the same method (115 μm for P0 and 140 μm for P2_5). The elastomers were pre-stretched (∼50%) by hand and attached on a round-shaped plastic frame (inner diameter: 2 cm, outer dimeter: 2.5 cm). Then, both sides of the elastomer film were covered by two Teflon-treated PET films with holes (diameter: 1 cm) in the middle. Both sides of the uncovered area were sputter-coated with silver (10 nm). After the electrodes were sputter-coated on both sides, the Teflon-treated PET films were removed, and the prepared actuators were connected to a high-voltage supply (PS375, Stanford Research system, US), following which voltage was applied step-wisely (3–5 V per μm per step) until breakdown. During the actuation tests, the diameter of the electrodes was measured by a high-speed optical micrometer (TM-065T, Keyence, Japan). Area actuation was calculated as the ratio of the electrode area after expansion divided by the area before expansion.

## Results and discussion

### Preparation and recycling procedures of the DEA

**Fig. 1:**
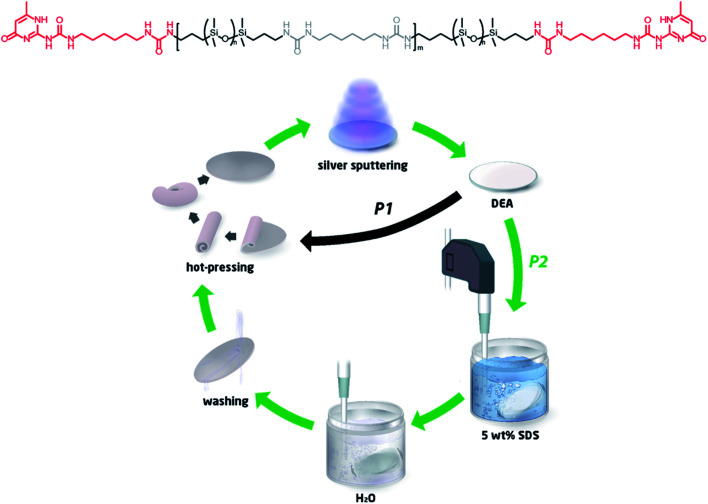
Recycling procedures for the TP-PDMS-based DEA (pathways 1 and 2). In both pathways, the TP-PDMS (urea linkages indicated in grey, UPys in red) was hot-pressed into a disc, which was sputter-coated with silver. In pathway 1, this was then directly returned to the first step and recycled back through the process. In pathway 2, the electrode was removed by ultra-sonication in SDS solution (5 wt%) for an hour, followed by ultra-sonication in deionised water for 10 minutes and an aqueous wash. Ultimately, this material was then returned to the first step and reused in the preparation of a new DEA.

Recently, we prepared a thermoplastic PDMS (TP-PDMS) elastomer based on the dual associative nature of a cross-linked network by urea and urea-adjacent ureidopyrimidone (UPy) units (number-averaged molecular weights (*M*_n_): 134 000 g mol^−1^, Fig. 1).^11^ The urea links connected approximately six linear PDMS segments (*M*_n_: 21 000 g mol^−1^), and both chain ends were end-capped with urea-UPys, thereby enabling laterally stacked structures of dimers between polymer end-groups. These cross-linked the main chain backbones strongly, while the urea group contributed with weak associations between the chains. The elastomer had a Young's modulus of 190 kPa and an ultimate elongation of 440% (at a strain rate of 30 mm min^−1^), therefore representing a typical material choice for a DEA.

The elastomer was used to fabricate a DEA, as illustrated in Fig. 1, which illustrates the initial preparation of the DEA followed by the recycling loop. First, the TP-PDMS was hot-pressed into a disc at a thickness of 1 mm and then sputter-coated with 10 nm silver electrodes (both sides) to produce a simple planar DEA. The recycling process was then investigated through two pathways (P1 and P2 in Fig. 1): either direct recycling of the material (P1) through roll milling and thermal reprocessing or through a simple washing and sonication process (P2), which was then followed by thermal reprocessing. The materials recycled through P1 are denoted herein as P1_1 and P1_2 for the once- and twice-recycled elastomers, without any cleaning. Direct recycling took place by roll-milling the whole DEA at 140 °C, to randomly distribute the silver electrode within the PDMS TPE. These samples act on this study as reference materials to elucidate the impact of residual silver in recycled samples. Washing and sonication were applied in the second pathway, whereby the prepared DEAs were ultra-sonicated in a 5 wt% SDS solution in water for an hour, in order to remove the majority of the silver electrodes. This was followed by short sonication in water for 10 minutes and then, finally, an aqueous rinse. The dried material was then directly inserted into the fabrication process for new DEAs. When the electrodes were removed from the DEAs, the samples were denoted as P2_1, P2_3, and P2_5 for 1, 3, and 5 times recycled elastomers, respectively.

### Accumulation of silver during the recycling cycles

**Fig. 2:**
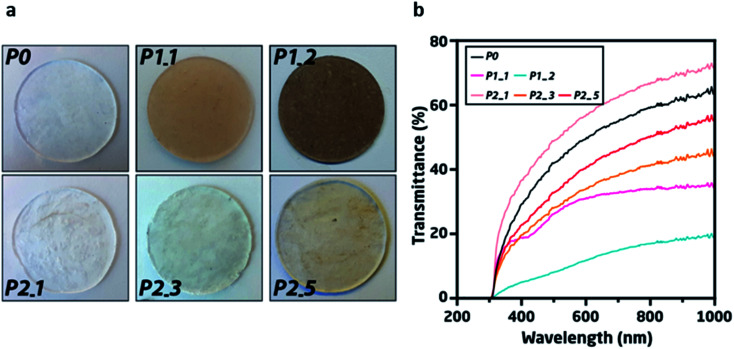
(a) Photographs of pristine elastomer (P0), directly recycled elastomers through pathway 1 (P1_1 and P1_2) and samples recycled through pathway 2 for 1, 3 and 5 times recycled elastomers (P2_1, P2_3 and P2_5). (b) UV-vis spectra of the pristine and recycled elastomers. The elastomer showed the strong absorption below 300 nm.

Without the cleaning procedures, DEA recycling resulted in an accumulation of silver within the samples, as was visually observed and also indirectly determined from the mechanical characterization (below). Although the relative added weight of silver to PDMS TPE was only ∼0.02 wt% per cycle, an obvious colour change can be seen for P1_1 and P1_2, when compared to the pristine material (P0) (top, Fig. 2a). Contrarily, no obvious colour change was observed on the samples recycled through P2, where the electrodes were removed from the DEAs during the recycling process. However, increased opaqueness was observed along with numbers of repeated recycling cycles with minor discolouration after the fifth cycle (bottom, Fig. 2a).

The accumulation of silver was observed by UV-vis spectroscopy, indicating minor absorbance at 420 nm in the UV-vis spectra^22^ and an overall reduction in transmittance for P1_1 and P1_2 (Fig. 2b). However, in the UV-vis spectra, only insignificant changes were observed for P2_1, P2_3, and P2_5, thereby demonstrating the effective removal of the majority of the silver during the five recycling phases.

### Evaluation of the effect of accumulated silver on dielectric elastomer properties

**Fig. 3:**
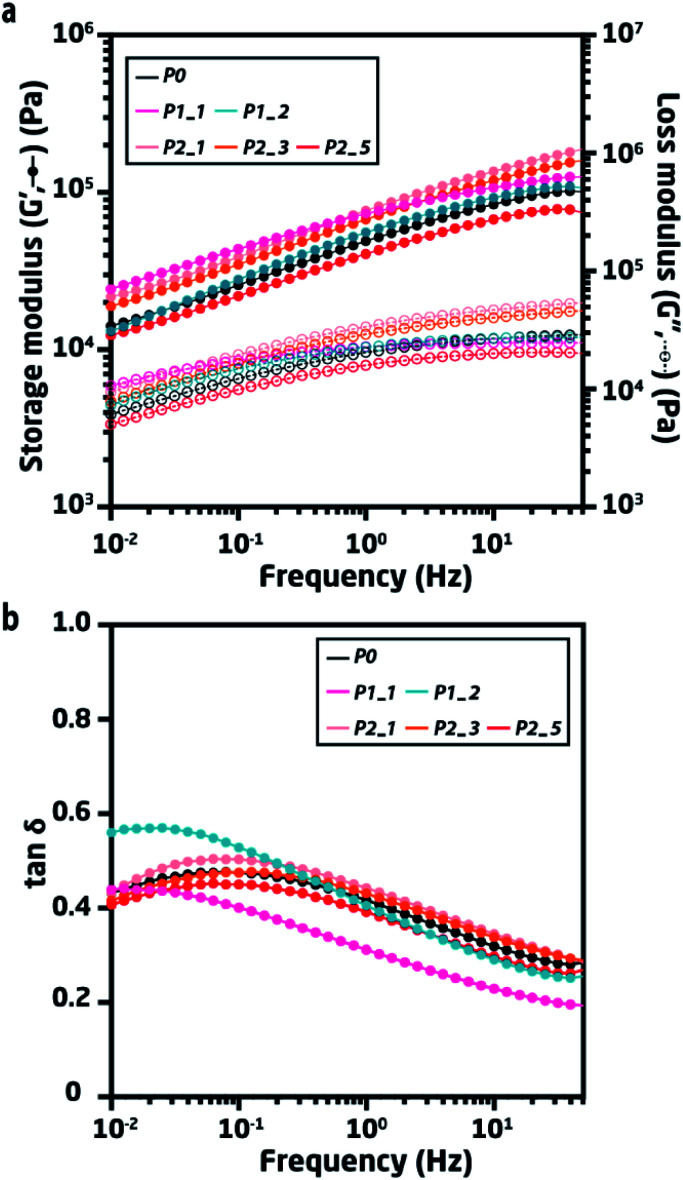
(a) Frequency sweeps of pristine and recycled elastomers at room temperature. (b) Rheological tan *δ* = *G*′′/*G*′ of pristine and recycled elastomers at room temperature.

The performance of DEAs is closely related to the mechanical properties of elastomers,^23^ and so any reduction in the mechanical properties of recycled materials resulting from impurities in the rinsing process, or from accumulated silver, is unacceptable. Rheometry was therefore used to observe the relaxation behaviours of the recycled materials, *i.e.* interactions between the polymer and silver can be determined, as well as changes in cross-linking density (Fig. 3).

Frequency sweeps show the frequency-dependent mechanical properties of the materials when under shear stress with different frequencies at a fixed strain. Such frequency-dependency arises from the dynamic strands of the networks, where PDMS chains undergo a sliding motion to achieve equilibrium under any given deformation. Tan *δ* is expressed as the ratio of *G*′′ and *G*′, which conveniently shows where the strongest relaxation occurs by the maximum. In the case of P0, the strongest relaxation was observed at 0.1 Hz.

Both P1_1 and P1_2 show slower relaxation behaviour (∼0.02 Hz) compared to P0, thus indicating an interaction between the polymer chains and silver (or other impurities) that slows down the rate at which the polymer chains relax. The accumulated silver not only slows the relaxation of the TP-PDMS, but it also broadened the tan *δ* curves for both P1_1 and P1_2, evidencing the dynamics of a more heterogeneous polymer. Moreover, the accumulated silver decreases tan *δ* for P1_1 for the investigated frequencies, indicating a more solid-like behavior due to the accumulated silver serving as fillers.

In comparison, P1_2 shows less interaction between the polymer chains and the silver, even though the sample contains twice as much silver as P1_1. The observed differences in tan *δ* for P1_1 and P1_2 highlight the effect of randomly distributed silver within the elastomer. The overall increase in tan *δ* of P1_2 compared to that of P1_1 may indicate that the accumulated silver partially hinders the hydrogen bonded network, while it still serves as a filler.

Conversely, the cleaned samples from pathway 2 (P2) all show similar linear viscoelastic properties compared to P0, thereby indicating an effective cleaning method where the network structure remains unaffected.

**Fig. 4:**
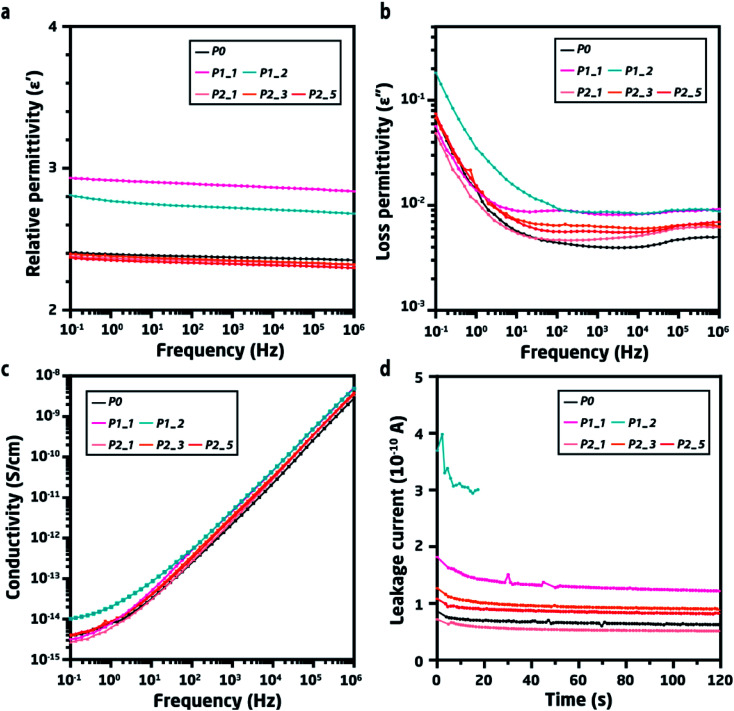
(a) Relative permittivity of pristine and recycled elastomers as a function of frequency at room temperature. (b) Loss permittivity of pristine and recycled elastomers at room temperature. (c) Electrical conductivity of pristine and recycled elastomers. The calculated applied electrical field for each sample is 3.5, 3.5, 3.0, 4.3, 3.1, and 2.3 V μm^−1^ for *R*_0_, P1, *R*_3_, *R*_5_, *R*_n1_, and *R*_n2_, respectively. (d) Leakage current of pristine and recycled elastomers at a fixed voltage (10 V μm^−1^) for 2 minutes. The measurement was stopped for *R*_n2_ within 20 seconds, since the sample underwent electrical breakdown.

Dielectric spectroscopy was used to investigate the effects of trace impurities and their resulting polarisation in the recycled materials' elastomer matrix. It is clear from relative permittivity (Fig. 4a) that the cleaned recycled materials maintained their original levels and experienced only a very minor increase in dielectric loss after five recycling cycles. Conversely, the directly recycled material had higher relative permittivity as well as a significant increase in loss permittivity (Fig. 4b), thus significantly deviating from the pristine material already within the first – and especially after the second – cycle. The presence of residual silver and the effect of its poor distribution can be also observed from the increase in loss permittivity (*ε*′′) for both P1_1 and P1_2. Furthermore, the dielectric loss (tan *δ*_d_ is illustrated in Fig. S1†) of P1_2 was higher than that of P0. Such higher overall loss can be explained by the increased loading of silver, whereby dispersed silver will result in a larger interfacial area.^24,25^

Various types of impurities will lead to increased permittivity, albeit this only indicates that foreign elements are present in the system. However, whether this would prevent direct recycling would depend on the impact on the system's electrical properties. Conductivity as a function of electric frequency, as well as the leakage current, was therefore determined for all samples (Fig. 4c). For the cleaned samples from pathway 2, there appear to be no observable differences in AC conductivity throughout the frequency range, which was also the trend observed from the DC conductivity (Fig. S2†). Conversely, the directly recycled sample after two cycles (P1_2) shows a significant increase in conductivity, and the emergence of a plateau in conductivity at low frequencies (<10^2^ Hz) can be observed, which indicates the creation of conductive pathways in the system.

This increase in conductivity for P1_2 was also the source of the observed increase in dielectric loss for P1_2 in the low-frequency region.^26^ Such changes in the dielectric properties of DEAs must be avoided, since this could lead to premature failure and lead to multiple breakdown mechanisms occurring simultaneously. In general, when one breakdown mechanism is active, it will accelerate others and ultimately lead to a destructive sequence of events.^27^ For instance, poorly distributed silver causes an inhomogeneous electric field, thereby generating non-equilibrium electrical stresses that cause electromechanical breakdown.^16,28^ Furthermore, DEA operation increases temperature,^29^ while observed dielectric loss contributes to increased heat generation by transferring mobile charges from silver to the polymer.^30^ As a result, the conductivity of the material will be greater, due to the temperature increase, thus resulting in Joule heating,^31^ insulation failure^16^ and, ultimately, electro-thermal breakdown. Yet, it must be noted that the breakdown mechanisms are still under discussion due to their complexity.

Consequently, if P1_1 and P1_2 are used for fabricating DEAs, the changes in dielectric properties (shown previously) lower the quality of devices, which in turn may experience premature failure. The quality of the DEAs can be evaluated by measuring their leakage current at a moderate voltage, which helps predict their possible untimely failure.^32^ Herein, the leakage current was measured at 10 V μm^−1^ for the pristine and recycled elastomers (Fig. 4d). Due to the accumulated silver, P1_1 had a higher leakage current than P0, while P1_2 had a more than four times higher leakage current until electrical breakdown terminated the measurement. This clearly shows that direct recycling is not a feasible approach, even though the TP-PDMS enables it. Comparatively, the properly cleaned samples (P2_1, P2_3, and P2_5) had only minor increases in leakage current, verifying that recycling with proper cleaning procedures can effectively remove silver electrodes from DEAs and still enable TP-PDMS to maintain its mechanical and dielectric properties.

It was therefore concluded that the material would be applicable for the fabrication of new DEAs – as corroborated through simple actuation tests on the silver sputtered samples. Direct electromechanical testing of the sputter coated samples shows a reduction of the overall potential of the samples, but nonetheless the simple actuation experiments confirm sufficient purity of the samples. Both the pristine and recycled materials (P0 and P2_5) demonstrated similar actuation behaviours (Fig. S3†), whereas P2_5 showed a larger actuation capabilities which may result from the residual silver causing a higher electrical field locally while also electro-mechanically stabilizing it. P0 underwent electrical breakdown at 21 V μm^−1^ during actuation, while P2_5 broke down at 18 V μm^−1^ (a 14% reduction compared to the pristine sample).

## Conclusions

A simple recycling process for a dielectric elastomer actuator based on a thermoplastic PDMS elastomer with silver electrodes was presented. The purification procedure permits the removal of the majority of electrode material through a simple washing step, where a surfactant wash is combined with a sonication process. By comparing the washing method with the corresponding direct recycling of the metal-coated thermoplastic elastomer, it became clear that directly recycling the material without the washing step is not a sufficiently effective pathway and clearly underlines the need to remove as much of the conductive elastomer as possible.

Specifically, the washing procedure was shown effective for up to five recycling phases, where generally very minor differences in material properties were observed. Rheological and dielectric behaviours were comparable to those of the pristine material, and only a minor reduction in performance was observed in terms of leakage current and electrical breakdown, in that the leakage current increased by 27% and electrical breakdown reduced by 14%. These minor changes in the properties of the recycled polymers can also be further suppressed by blending them into the pristine polymers, thereby suppressing the effects of recycling on the final DEA. In addition, the reclaimed polymers can be used for fabricating products that are not as high quality as DEAs. Effectively, this not only enables the recycling of elastomer material from production waste, but it also opens up the possibility of reclaiming material from used devices. In both cases, this will result in a reduction in both material consumption during production as well as in the amount of generated waste.

## Conflicts of interest

There are no conflicts to declare.

## Supplementary Material

RA-012-D2RA00421F-s001
