# Supplementary material for: Recycling of dielectric electroactive materials enabled through thermoplastic PDMS
Source: RSC Adv. 2022 Mar 16;12(14):8449–57. doi: 10.1039/d2ra00421f (PMC8984943; doi:10.1039/d2ra00421f)
Supplement: RA-012-D2RA00421F-s001 [file RA-012-D2RA00421F-s001.pdf]

Electronic Supplementary Material (ESI) for RSC Advances.
This journal is © The Royal Society of Chemistry 2022

Supporting information

Recycling of dielectric electroactive materials enabled through thermoplastic PDMS

S. Jeong, A.L. Skov, A.E. Daugaard*

Danish Polymer Centre, Department of Chemical and Biochemical Engineering, Building 227

Technical University of Denmark, 2800 Kgs. Lyngby, Denmark

* adt@kt.dtu.dk

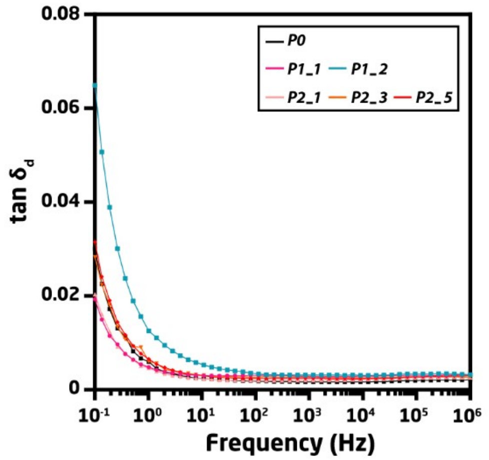

Figure S1. Dielectric loss tangent (tan δ_d) of pristine and recycled elastomers.

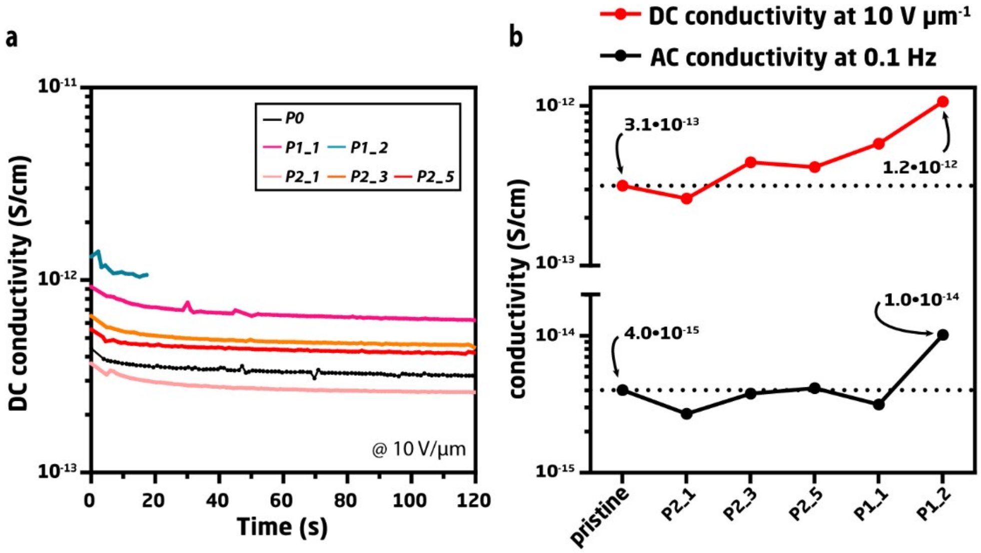

Figure S2. (a) DC conductivity of pristine and recycled elastomers from the leakage current tests at 10 V μm^{-1}. (b) Comparison between AC conductivity (black) and DC conductivity (red) of pristine and recycled elastomers. The DC conductivity of all samples was obtained 120 seconds after the electrical

field was applied from the leakage current tests. In the case of Rn_2, the value of conductivity before the breakdown was taken.

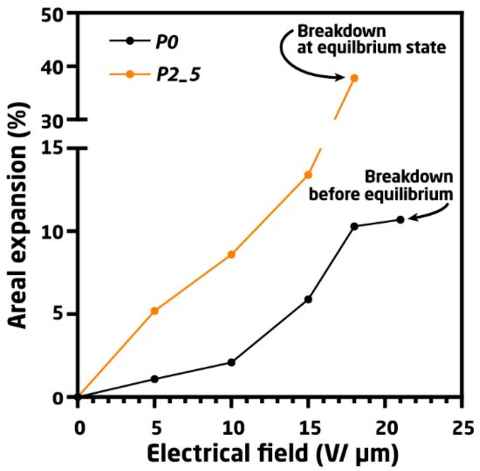

Figure S3. Actuation of pristine (P0) and recycled elastomer (P2_5, orange). For each step, a voltage was applied for approximately 10 seconds, and then the given voltage was removed for another 10 seconds while the electrode area was measured. During the measurements of the electrode area, the maximum value was taken, and the areal expansion ($\Delta A/A_0 \times 100(\%)$) was calculated.
